# Identification in *Marinomonas mediterranea* of a novel quinoprotein with glycine oxidase activity

**DOI:** 10.1002/mbo3.107

**Published:** 2013-07-22

**Authors:** Jonatan Cristian Campillo-Brocal, Patricia Lucas-Elio, Antonio Sanchez-Amat

**Affiliations:** 1 Department of Genetics and Microbiology, University of MurciaMurcia, 30100, Spain

**Keywords:** Glycine oxidase, hydrogen peroxide, *Marinomonas mediterranea*, quinoprotein.

## Abstract

A novel enzyme with lysine-epsilon oxidase activity was previously described in the marine bacterium *Marinomonas mediterranea*. This enzyme differs from other l-amino acid oxidases in not being a flavoprotein but containing a quinone cofactor. It is encoded by an operon with two genes *lodA* and *lodB*. The first one codes for the oxidase, while the second one encodes a protein required for the expression of the former. Genome sequencing of *M. mediterranea* has revealed that it contains two additional operons encoding proteins with sequence similarity to LodA. In this study, it is shown that the product of one of such genes, Marme_1655, encodes a protein with glycine oxidase activity. This activity shows important differences in terms of substrate range and sensitivity to inhibitors to other glycine oxidases previously described which are flavoproteins synthesized by *Bacillus*. The results presented in this study indicate that the products of the genes with different degrees of similarity to *lodA* detected in bacterial genomes could constitute a reservoir of different oxidases.

## Introduction

l-amino acid oxidases (LAOs) are enzymes that are able to catalyze the oxidative deamination of amino acids, generating a keto acid, ammonia, and hydrogen peroxide. The generation of the last compound confers to them antimicrobial properties (Barsby 2006). Most of the LAOs are flavoproteins and are therefore characterized by the presence of a flavin adenine dinucleotide (FAD)-binding domain (Dym and Eisenberg 2001). As far as we know, the only described oxidase acting on l-amino acids that is not a FAD protein is the lysine oxidase (LodA) (EC 1.4.3.20) synthesized by *Marinomonas mediterranea* which contains a protein-derived quinone cofactor (Gomez et al. 2010). Quinone cofactors are generated by posttranslational modification of amino acid residues. Pyrroloquinoline quinone (PQQ) was the first quinone cofactor described (Salisbury et al. 1979). Although PQQ is a soluble cofactor, other known quinone cofactors are nondissociable, as they are generated by posttranslational modification of amino acid residues in the protein (Davidson 2007). LodA catalyzes the oxidation of l-lysine at the epsilon position of the side chain generating as products 2-aminoadipate 6-semialdehyde, ammonium, and hydrogen peroxide (Gomez et al. 2006). On the contrary, the other LAOs, such as the l-lysine-α-oxidase synthesized by *Trichoderma viride*, oxidize the amino group in the alpha position (Kusakabe et al. 1980).

LodA is encoded by the *lodAB* operon that contains, in addition to the *lodA* gene, a second gene, named *lodB,* encoding a protein required for the correct expression of LodA (Gomez et al. 2010). BLAST analysis revealed that proteins similar to LodA can be detected in many different bacteria (Lucas-Elio et al. 2006). In some of those bacteria, it has been shown that LodA homologs play a role, mediated by hydrogen peroxide, in biofilm differentiation and dispersal (Mai-Prochnow et al. 2008). In the case of the highly similar protein AlpP from *Pseudoalteromonas tunicata*, lysine oxidase activity was demonstrated (Mai-Prochnow et al. 2008). However, the actual enzymatic activity of other proteins with different levels of similarity to LodA remains to be determined. According to that, the genes encoding those proteins are generally annotated in bacterial genomes as encoding hypothetical proteins.

Recently, genome sequencing of *M. mediterranea* revealed that this microorganism possesses two genes, Marme_2396 and Marme_1655, similar to *lodA* (Lucas-Elio et al. 2012). The characterization of a *M. mediterranea* mutant strain with *lodAB* deletion indicated that it lacks lysine oxidase activity (Gomez et al. 2010), suggesting that those genes must be encoding a different enzyme. The characterization of the products of those genes is of broad interest as it may help to understand the role of the other genes similar to *lodA* detected in many different bacterial genomes. In this study, it is shown that Marme_1655 encodes a novel enzyme with glycine oxidase (GO) activity that has been named GoxA. This result indicates that proteins similar to *M. mediterranea* LodA and GoxA may constitute a reservoir of novel enzymatic activities.

## Experimental Procedures

### Strains, plasmids, primers, and culture media

The bacterial strains, plasmids, and primers used in this study are listed in Table 1. *Marinomonas mediterranea* strains were incubated in liquid medium at 25°C and 130 rpm, and were usually grown in marine broth and Marine Agar 2216 (Difco, Sparks, MD). Another medium used was Complex Marine Medium (MMC) (Solano et al., 1997) and the chemically minimal medium New Medium with Glucose and Lysine (MNGL) (Molina-Quintero et al., 2010). *Escherichia* was grown in Luria–Bertani (LB) medium at 37°C. When required, media were supplemented with the appropriate antibiotic (Sigma-Aldrich, St. Louis, MO).

**Table 1:** Bacterial strains, plasmids, and primers used in this work

Strain, plasmid, or primer	Relevant genotype and description, or sequence	Reference
Strains		
*Marinomonas mediterranea*		
MMB-1R, wild type	MMB-1, Rif^r^ spontaneous	Solano et al. (2000)
LD, *lodAB* mutant	MMB-1R, Δ*lodAB*	Gomez et al. (2010)
LGD, *lodAB*, and *goxAB* mutant	LD, Δ*goxAB*	This study
LGDAB	LGD Ω mini-Tn*10* Km^r^ (*goxAB*)	This study
*Escherichia coli*		
UM202	MP180 *katG*::Tn*10*	Loewen et al. (1985)
S17-1 (λ*pir*)	thi pro hsd (r^−^m^+^) *recA*::RP4-2-Tc^r^::Mu Km^r^::Tn7 Tp^r^ Sm^r^, lysogenized by *λpir* phage	de Lorenzo and Timmis (1994)
Plasmids		
pEX18Gm	Gm^r^;*oriT*^+^ *sacB*^+^, gene replacement vector with MCS from pUC18	Hoang et al. (1998)
pEX18GmGD	pEX18Gm with a *Sac*I-*Bam*HI 818-bp fragment from upstream operon *goxAB* and a 787-bp *Bam*HI-*Pst*I fragment from downstream the operon *goxAB*, both cloned in its MCS.	This study
pBSL182	*ori* R6K, *mob* RP4, Ap^r^; mini-Tn*10* Gm^r^	Alexeyev and Shokolenko (1995)
pBlodA II	*ori* R6K, *mob* RP4, Ap^r^; mini-Tn*10* Km^r^, *lodA*	Gomez (2010)
pBgoxAB	*ori* R6K, *mob* RP4, Ap^r^; mini-Tn*10* Km^r^, *goxAB*	This study
Primers^1^		
GOXDIRSAC	5′-ACGCTTTGGAGCTCATACTACTG-3′	
GOXREVBAM	5′-CGTCCTATCTGGATCCATTAATATGAAA-3′	
GOXDIRBAM	5′-CGCACAAGGATCCCTAACGGTTTC-3′	
GOXREVPST	5′-TATAGGGAGAACTGCAGGGGAAAAC-3′	
GOSEC1	5′-GAAATCCCACCGGTTACAAC-3′	
GOREVSEC2	5′-CCTCGGAGTTTGGACGTTG-3′	
pGODIRKPN1	5′-TACACTTCAGGTACCTTCCTTATACAAC-3′	
GOREVPST1	5′-CATTATCCGTTTTGACCTGCAGAGTGG-3′	
GODIRNDE1	5′-GATAGGACGATCATATGCAAAATGACGG-3′	
GOREVSMA1	5′-GTTTTGACCTACACCCGGGTTAATTGATG-3′	

1 Restriction sites are underlined.

### Detection and concentration of GOX from *M. mediterranea* LD supernatants

*Marinomonas mediterranea* strains were grown at 25°C and 130 rpm in MNGL from an initial inoculum with OD_600_ = 0.05. After 48 h, the cultures were harvested by centrifugation at 5000*g* for 10 min and the supernatant obtained was considered the extracellular fraction. In order to concentrate the GOX activity for enzymatic measurements, two volumes of ethanol were added to the extracellular fraction and left overnight at −20°C. The suspension was centrifuged at 25,000*g* at 4°C for 10 min and the pellet obtained was then allowed to air dry and resuspended in 1/10 volume, compared to the initial supernatant, of 50 mmol/L sodium phosphate buffer, pH 7.4 with NaCl 500 mmol/L. On the other hand, supernatants of *M. mediterranea* submitted to SDS-PAGE (sodium dodecyl sulfate polyacrylamide gel electrophoresis) (see below) were concentrated 100× by using Amicon® Ultra centrifugal filters 30K (Millipore-Merck, KGaG, Darmstadt, Germany).

### Antibiogram assays

Antibacterial activity due to Gox was assayed through antibiograms. A suspension of *E. coli* UM-202 (Loewen et al. 1985) in NaCl 0.85% (OD_600_ = 0.2) was seeded on LB plates. In some experiments 20 μL of concentrated supernatants were loaded into 6 mm disks of Filter Paper Backing (BioRad, Hercules, CA) and allowed to air dry before placing them onto the agar plate. To determinate the antimicrobial activity of proteins run by nondenaturing SDS-PAGE the protocol previously described was used (Lucas-Elio et al. 2005). After fixing and washing, the gel was sliced and placed onto the antibiogram plate. Antibiograms plates were incubated for 48 h at 25°C.

### Glycine oxidase activity assays

#### Fluorimetric determination of H_2_O_2_ production

To detect the GOX activity a fluorimetric assay was routinely used (Amplex Red hydrogen peroxide/peroxide assay; Invitrogen; Gomez et al. 2006). The assay mixture (100 μL) contained 2 or 20 mmol/L of the substrate in 0.05 mol/L of sodium phosphate buffer with NaCl 0.5 mol/L pH 7.4, 0.05 mmol/L Amplex Red, 0.1 U/mL of peroxidase, and 10 μL of sample. Reactions were carried out at 37°C for 15 min in 96-well ELISA (enzyme-linked immunosorbent assay) plates. Amplex Red oxidation was followed using an excitation filter of 550 nm and an emission filter of 590 nm in a FLUOstar Optima (BMG LabTech, Ortenberg, Germany). Background fluorescence due to the slow spontaneous oxidation in the absence of glycine was subtracted. Activities were normalized according to the milligrams of protein present in each sample, measured by the Bradford assay (Sigma).

To estimate the oxidase activity of LD strain extracellular fraction on different compounds, the fluorimetric assay was performed as described before. In order to evaluate the effect of different inhibitors on GOX activity, supernatants of *M. mediterranea* were preincubated with 1 mmol/L semicarbazide, 100 μmol/L hydroxylamine, 100 μmol/L phenylhydrazine, or 100 μmol/L methylhydrazine for different times. Ten microliters of those samples were used for the fluorimetric assay, what constitutes a 10× dilution of the inhibitor. In these experimental conditions, phenylhydrazine strongly inhibited horseradish peroxidase (HRP), so after incubation, those samples were subjected to dialysis against sodium phosphate buffer, using 10 mm/10 mL Spectra/Por Float-A-Lyzer (Spectrum Laboratories, Breda, the Netherlands) dialysis membrane tubes (molecular weight cutoff = 1500), in order to remove most of the inhibitor before enzymatic measurements.

The fluorimetric assay was also used to determine directly GOX activity in slices cut from SDS-PAGE which were previously fixed and washed with deionised water (see below in SDS-PAGE).

#### Ammonium determination by the glutamate dehydrogenase-coupled assay

This method is based on the detection of ammonium by a coupling reaction with the glutamate dehydrogenase according to the following reaction:

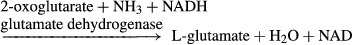


The decrease in absorbance at 340 nm due to the oxidation of NADH cofactor was followed (Job et al. 2002). The reaction was carried out for 15 min at 37°C in 384-well plate with 5 mmol/L 2-oxoglutarate, 0.25 mmol/L NADH, 20 U/mL glutamate dehydrogenase from Bovine Liver (Sigma-Aldrich), glycine 2 mmol/L, and 10 μL of sample, in 0.05 mol/L of sodium phosphate buffer pH 7.4. The loss of absorbance due to spontaneous autoxidation of NADH was subtracted for each test by performing the pertinent controls.

#### SDS-PAGE

SDS-PAGE was carried out by the method of Laemmli (1970) in nondenaturing conditions for *M. mediterranea* Gox with 3% acrylamide in the stacking gel and 8% acrylamide in the separating gel. Running buffer was 0.3% Tris, 1.44% glycine, and 0.1% SDS, pH 8.3. The samples were mixed with 1/2 volume of loading buffer containing 3 mol/L 2-mercaptoethanol, 0.18 mol/L Tris-HCl pH 6.8, 15% glycerol, 0.075% bromophenol blue, and 9% SDS. Gels were fixed for 2 h in a solution of 10% acetic acid and 20% isopropanol and washed for 2 h in deionised water in order to detect the antibacterial activity of the bands (Bhunia et al., 1987). Duplicate lanes charged with identical samples were treated separately and stained with Coomassie brilliant blue R250. According to the position of the stained bands, the duplicate nonstained lanes were cut in different slices and then were placed onto a seeded LB plate to see their antimicrobial effect, or into 96-well ELISA plates to measure GOX activity.

### Mass spectrometry analysis

#### In-gel trypsin digestion

Samples were digested with the following standard procedure. SDS-PAGE gel was stained using PageBlue Coomassie Protein Staining Solution (Thermo Fisher Scientific, Rockford, IL) and selected spots were spliced in approximately 2 × 2 mm bands. After being destained, the spots were washed twice with MilliQ distilled water and then twice with 25 mmol/L ammonium bicarbonate buffer pH 8.5 in 50% acetonitrile during 30 min at 37°C. After removing the supernatant, spots were dried for 15 min using an Eppendorf 5301 vacuum evaporator, and then they were incubated with 50 μL of 25 mmol/L ammonium bicarbonate buffer pH 8.5 with 20 mmol/L DTT (Sigma-Aldrich) at 56°C for 20 min. The supernatant was removed and samples were alkylated by adding 25 mmol/L ammonium bicarbonate buffer pH 8.5 with 100 mmol/L iodoacetamide (Sigma-Aldrich) during 30 min at room temperature in the dark. The supernatant was again removed and spots were washed first with 25 mmol/L ammonium bicarbonate buffer pH 8.5 and then with 25 mmol/L ammonium bicarbonate buffer pH 8.5 in 50% acetonitrile during 15 min at 37°C each time. After washing, spots were dried again and then incubated with 25 mmol/L ammonium bicarbonate buffer pH 8.5 containing 0.01% ProteaseMax (Promega, Madison, WI) and 0.25–0.5 μg of Trypsin Gold Proteomics Grade (Promega) during 45 min at 4°C and finally submitted to digestion during 16 h at 37°C. ProteaseMax is a surfactant product that enhances the trypsin digestion. The supernatant was collected in a new tube, and the spots were washed with 50 μL of a solution containing 50% acetonitrile and 0.5% trifluoroacetic acid (TFA) and then with 50 μL of acetonitrile during 30 min at 37°C each time. These washes enhanced the extraction of digested fragments from the gel spots and both supernatants after washing were collected in the same tube and dried using a vacuum evaporator.

#### HPLC-MS/MS analysis

The separation and analysis of the tryptic digests of the samples were performed with a HPLC-MS (high-performance liquid chromatography mass spectroscopy) system consisting of an Agilent 1100 Series HPLC (Agilent Technologies, Santa Clara, CA) equipped with a micro-well plate autosampler and a capillary pump, and connected to an Agilent Ion Trap XCT Plus Mass Spectrometer (Agilent Technologies, Santa Clara, CA) using an electrospray (ESI) interface. Experimental parameters for HPLC were set in Chemstation software (Agilent Technologies, Rev. B.01.03), while Ion Trap parameters were set in LC/MSD Trap Control Software (Bruker Daltonik, v5.3, Germany).

Dry samples from in-solution or in-gel digestions were resuspended in 10 μL of buffer A, consisting in water/acetonitrile/formic acid (94.9:5:0.1). Sample was injected onto a Zorbax SB-C18 HPLC column (5 μm, 150 × 0.5 mm, Agilent Technologies, Santa Clara, CA), thermostated at 40°C, at a flow rate of 10 μL/min. After the injection the column was washed with buffer A for 30 min and the digested peptides were eluted using a linear gradient 0–80% B (buffer B: water/acetonitrile/formic acid, 10:89.9:0.1) for 120 min. The column was washed with the initial conditions (buffer A) for 30 min before the following sample injection. The column was coupled online to an Agilent Ion Trap XCT Plus mass spectrometer using an electrospray interface.

The mass spectrometer was operated in the positive mode. The nebulizer gas pressure was set to 15 psi, whereas the drying gas was set to a flow of 5 L/min at a temperature of 350°C. The capillary spray voltage was 3500 V, whereas the scan speed was set to 8100 (m/z)/sec from 200 to 2200 m/z, with a target mass of 1000 m/z, and 3 spectra averaging. Smart ion target was set to 150,000, while the maximum accumulation time was 50 msec. MS/MS data were collected in an automated data-dependent mode (AutoMSn mode). The three most intense ions were sequentially fragmented using helium collision-induced dissociation (CID) with an isolation width of 2 and a relative collision energy of 35%. The same ion was rejected after two consecutive scans.

Data processing was performed with DataAnalysis program for LC/MSD Trap Version 3.3 (Bruker Daltonik, GmbH, Germany) and Spectrum Mill MS Proteomics Workbench (Rev A.03.02.060B, Agilent Technologies, Santa Clara, CA).

Briefly, raw data were extracted under default conditions as follows: unmodified or carbamidomethylated cysteines; sequence tag length >1; [MH]+ 50–7000 m/z; maximum charge +7; minimum signal-to-noise (S/N) 25; finding ^12^C signals. The MS/MS search against protein sequences of interest was performed with the following criteria: identity search mode; tryptic digestion with three maximum missed cleavages; carbamidomethylated cysteines; peptide charge +1, +2, +3; peptide precursor mass tolerance 2.5 Da; product ion mass tolerance 0.7 amu; ESI ion trap instrument; minimum matched peak intensity 50%.

### DNA manipulations

DNA was manipulated according to standard protocols (Sambrock and Rusell 2001). KOD DNA polymerase (Merck KGaG) was used for polymerase chain reaction (PCR). Restriction enzymes were purchased from Fermentas (Thermo-Fischer Scientific). DNA restriction fragments were eluted from agarose gels by utilizing Qiaquick columns (Qiagen, Hilden, Germany). T4 DNA ligase was from Invitrogen. Transformation of electrocompetent cells of *E. coli* S17-1λpir was carried out by electroporation (Dower et al. 1988). Isolation of plasmid DNA from *E. coli* was achieved by the Wizard Plus SV Minipreps DNA Purification System from Promega. To test the constructions, chromosomal DNA was isolated with Wizard kit from Promega.

### Construction of suicide plasmids for deletion

The first step toward obtaining the mutant strains was the cloning into the suicide plasmid pEX18Gm (Hoang et al. 1998) of upstream and downstream DNA sequences flanking the genomic region to be deleted. This plasmid has the multiple cloning site (MCS) of pUC18, contains the counterselectable *sacB* marker and an *oriT* for conjugation-mediated plasmid transfer.

To create the vector pEX18GmGD for the deletion of genes Marme_1655 and Marme_1654, we cloned the upstream 818 bp (α fragment), product of digestion with *Sac*I and *Bam*HI of the fragment amplified by PCR with primers GOXDIRSAC-GOXREVBAM. The downstream 787-bp fragment for this plasmid (β fragment) was a *Bam*HI-*Pst*I digestion of the PCR fragment generated using primers GOXDIRBAM-GOXREVPST (Fig. S1).

### Insertion of the deletion cassette into the genome followed by plasmid excision to generate the deletion

Plasmid pEX18GmGD was electroporated into *E. coli* S17-1λpir for conjugation into *M. mediterranea* LD strain by the mating method described in other works (Solano et al. 2000). Transconjugants containing single crossover of each plasmid integrated into the genome were selected on MMC or 2216 agar containing rifampicin (Rif) 50 μg/mL and gentamcin (Gm) 10 μg/mL. Correct insertion of the plasmids was confirmed by PCR.

Single crossover strains were grown in MMC at 25°C and 130 rpm for 16 h in the absence of Gm to enrich for cells in which a second crossover event had occurred. Then several dilutions of those cultures were plated on Complex Marine Medium with 5% Sucrose (MMCS) agar and incubated at 25°C for 2 days. Sucrose resistant colonies were reinoculated on MMCS agar and screened for loss of gentamicin resistance encoded by the vector. PCR analysis confirmed that the mutants isolated from some colonies had undergone excision of pEX18Gm, resulting in unmarked deletion strains. Therefore, PCR with internal primers of the *gox* operon GOSEC1-GOREVSEC2 was carried out to confirm the absence of the operon. To check that deletion had taken place, we used external primers of the *gox* operon GOXDIRSAC-GOXREVPST under short extending times in which a PCR product will not appear in the undeleted strains and only will appear provided the deletion had happened.

### Genetic complementation of LGD mutant strain

For genetic complementation of LGD mutant strain, the genes in *gox*AB operon were cloned in the MCS of mini-Tn*10* transposon in pBSL182 vector (Alexeyev and Shokolenko 1995). We amplified the *goxAB* operon from wild type with an upstream 210 bp containing the promoter region using pGODIRKPN1 and GOREVPST1 primers. This PCR product was cloned in the suicide plasmid pBlodAII derivative of pBSL182 vector (Gomez et al. 2010, Doctoral thesis) using the *Kpn*I and *Pst*I restriction sites in the primers, and obtaining the pB*goxAB* plasmid. This plasmid was mobilized from donor strain S17-1(λpir) into LGD mutant strain by conjugation and the kanamycin resistant strains obtained were PCR checked for transposon insertion.

## Results

### Screening of oxidase activities in *M. mediterranea* LD

**Figure 1:**
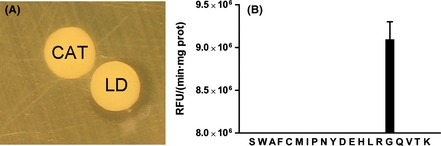
Antimicrobial and oxidase activity in *Marinomonas mediterranea*LD (*ΔlodAB*) supernatants. (A) Antibiograms in LB medium against *Escherichia coli* UM202. LD, disk loaded with 20 μL of 10× concentrated supernatant; CAT, disk loaded with 20 μL of catalase 10 mg/mL. (B) Oxidase activity against the proteinogenic amino acids at a concentration of 2 mmol/L measured using the fluorimetric assay for hydrogen peroxide detection. Activity is expressed as relative fluorescence units (RFU) per min and mg of protein.

*Marinomonas mediterranea* LodA shows lysine oxidase activity. As this activity is not detected in mutant strain LD (Δ*lodAB*) (Gomez et al. 2010), it was hypothesized that the two operons similar to *lodAB* detected in the *M. mediterranea* genome (Lucas-Elio et al. 2012), could encode different oxidases. In a preliminary screening, an antimicrobial activity was observed in *M. mediterranea* LD supernatants. The inhibition by catalase of that activity suggested that hydrogen peroxide was involved (Fig. 1A). Later, using a fluorimetric assay it was confirmed the generation of hydrogen peroxide when glycine was used as substrate. The activity was specific for this amino acid, as no other proteogenic amino acid could be oxidized (Fig. 1B). The enzyme GO (EC 1.4.3.19) has been previously described in microorganisms of the genera *Bacillus* (Nishiya and Imanaka, 1998) and *Geobacillus* (Martínez-Martínez et al. 2008). GO is a flavoprotein that catalyzes the oxidative deamination of glycine generating glyoxylate, hydrogen peroxide, and ammonium. GO shows similarity in sequence and substrate range with other flavoproteins such as d-amino acid oxidase (DAAO, EC 1.4.3.20) and sarcosine oxidase (SOX, EC 1.5.3.1), although this last enzyme is not able to oxidize glycine and the former only shows low activity on that aminoacid (Molla et al. 2003). To check if *M. mediterranea* could encode a typical GO, its genome was explored using as query for BLAST analysis the sequence of the *Bacillus subtilis* GO (Thi0, accession BSU11670). The most similar protein, showing 24.9% identity and 39.5% similarity, was the product of Marme_2428. However, this protein showed higher identity (43.3%) and similarity (58.8%) to the beta-subunit of the heterotetrameric SOX from *Corynebacterium* sp. U-96 (accession number BAD97816.1). The possibility that Marme_2428 encodes a SOXs is also supported by the observation that it forms part of a cluster of genes (Marme_2425 to Marme_2429) with similarity to genes coding for α, β, δ, and γ SOX subunits. BLAST using as a query the sequence of monomeric SOX from *Bacillus* (accession number P40859) and the DAAO from *Rhodotorula gracilis* (accession number P80324) gave no hits with an expected value higher than 1 e^−5^. In conclusion, the bioinformatic analysis did not reveal any gene that might encode a GO similar to those previously described.

### Preliminary characterization of *M. mediterranea* GO in comparison with other oxidases

The oxidation of glycine by an oxidase would release, apart from hydrogen peroxide, other compounds such as glyoxylate and ammonium. The production of ammonium was detected spectrophotometrically by the method of the glutamate dehydrogenase-coupled assay. By using this method it was observed an specific activity in the supernatants of *M. mediterranea* LD of 0.076 U/mg of protein. The Amplex Red hydrogen peroxide/peroxidase assay used in the fluorimetric assays can also be used to calculate International Units measuring the generation of hydrogen peroxide. This compound, in the presence of peroxidase, reacts with the Amplex Red reagent in a 1:1 stoichiometry to produce the red-fluorescent oxidation product, resorufin which has a high extinction coefficient (58,000 ± 5000 cm^−1^ M^−1^). Using this method, 0.057 U/mg protein was detected in the supernatants, which is in good agreement with the calculation of ammonium generation.

The *M. mediterranea* GO was further characterized in terms of some biochemical properties in comparison with other GOs previously described in *B. subtilis* (Nishiya and Imanaka 1998) and *Geobacillus kaustophilus* (Martínez-Martínez et al. 2008). The analysis of the substrate range revealed that the *M. mediterranea* enzyme was more specific for glycine than any of the *Bacillus* or *Geobacillus* enzymes (Table 2). In fact, those enzymes showed a higher activity on other substrates such as sarcosine (*B. subtilis*) or d-proline (*G. kaustophilus*) than on glycine. On the contrary, the *M. mediterranea* enzyme only showed 0.1% and 0.3% activity, respectively, on those two substrates. On the other hand, the Km for the *M. mediterranea* GO was 8.33 mmol/L which is higher than the value previously reported for the oxidation of glycine by the *Bacillus* and *Geobacillus* enzymes (0.22–0.99 mmol/L) (Nishiya and Imanaka 1998; Martínez-Martínez et al. 2008).

**Table 2:** Comparison of the relative *V*_max_ of different glycine oxidases against several substrates

	Gly	Sarcosine	Glycine ethyl esther	N-Ethylglycine	d-HPG	d-Ala	d-Pro	d-Glu	d-Lys
*Marinomonas mediterranea* Gox^1^	100	0.1	30.7	0.3	0.6	0.2	0.3	0.2	0.2
*Bacillus subtilis* GoxB^2^	77.4	100	NA	85.3	NA	7.4	15.1	ND	ND
*Geobacillus kaustophilus* GoxK^3^	69	36	22.3	22.9	NA	28	100	NA	NA

Activity is expressed as percentage of activity with respect to the best substrate of each enzyme. d-HPG, d-*p*-hydroxyphenylglycine; NA, not assayed; ND, not detected.

1 Supernatants of strain *M. mediterranea* LD against different substrates at 20 mmol/L concentration.

2 Data from Nishiya and Imanaka (1998).

3 Data from Martínez-Martínez et al. (2008).

**Figure 2:**
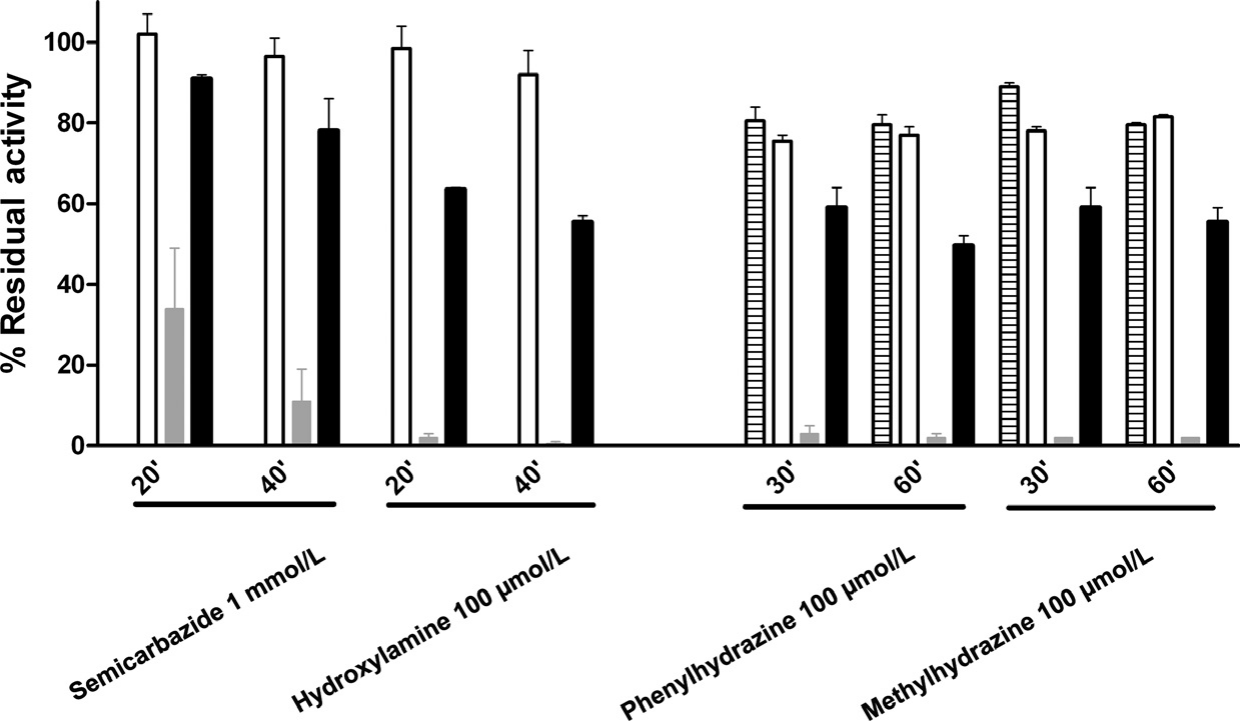
Sensitivity of *Marinomonas mediterranea*GOX (filled bars) to quinoprotein inhibitors. Controls: lysine-α-oxidase, a flavoprotein (empty bars), *Marinomonas mediterranea* LodA, a quinoprotein (gray bars), and horseradish peroxidase (HPR, stripped bars). The effect of remaining phenylhydrazine and methylhydrazine on HRP was determined by using 10 μmol/L H_2_O_2_ as substrate for the reaction.

The effect of several quinoprotein inhibitors was assayed on *M. mediterranea* GO activity. In this study *M. mediterranea* lysine-ε-oxidase, LodA, as a control of a quinoprotein (Gomez et al. 2010) and a commercial lysine-α-oxidase (LαO), as a control of a flavoprotein were included. As the fluorimetric assay is a coupled assay with HRP, the possible effect on this last enzyme was also assayed. It was observed that at the concentrations used, phenylhydrazine inhibited HRP, so after incubation with this inhibitor, the samples had to be dialyzed before enzymatic determinations (Fig. 2). As expected, the flavoprotein LαO was not inhibited by any of the compounds assayed, as the small effect of phenylhydrazine and methylhydrazine can be attributed to HRP inhibition. On the contrary, although the effect was not as strong as the inhibition observed in the case of LodA, the GO was sensitive to the quinoproteins inhibitors (Fig. 2).

In conclusion, the characterization of the *M. mediterranea* GO revealed that it showed important differences with previously described GOs and suggests that this activity is not due to a flavoprotein with amino acid oxidase activity. The next experiments were aimed at cloning the gene encoding this activity and to explore the possibility that this enzyme was encoded by one of the operons similar to *lodAB* detected in the *M. mediterranea* genome.

### Identification of the gene encoding the GO activity

**Figure 3:**
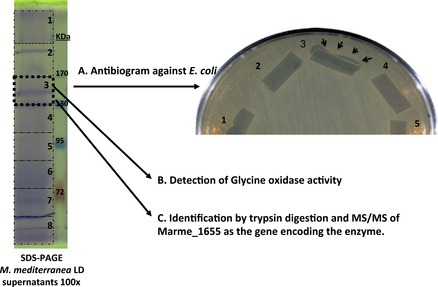
Identification of Marme_1655 as the gene encoding Gox. 100× concentrated supernatants of *Marinomonas mediterranea*LD (*ΔlodAB*) were run in SDS-PAGE under nondenaturing conditions. The gel was sliced in different fragments. The fragment between, approximately, 130–170 kDa showed antimicrobial activity, glycine oxidase activity, and several peptides matching the product of Marme_1655.

**Figure 4:**
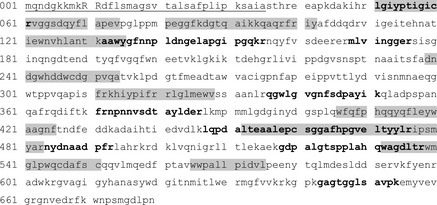
Sequence analysis of the product of Marme_1655. Bold letters indicate peptides detected after trypsin digestion and HPLC-MS/MS analysis. Highlighted sequences indicate conserved regions detected also in LodA and similar proteins (Lucas-Elio et al. 2006). Underlined sequence corresponds to the signal peptide with the twin-arginine motif (capital letters) predicted according to the TatP 1.0 server (Bendtsen et al. 2005).

Concentrated supernatants of *M. mediterranea* LD (*ΔlodAB*) where run under SDS-PAGE in the nondenaturing conditions previously described for LodA (Lucas-Elio et al. 2005). In those conditions a band with antimicrobial activity was detected (Fig. 3). In a set of parallel lines it was observed that in the approximately same region GO activity could be detected (data not shown). In addition, the band was excised from another gel line, trypsin digested, subjected to HPLC-MS/MS and analyzed against *M. mediterranea* genome. It was observed that several peptides matched the predicted product of the gene Marme_1655 (Fig. 4). This gene forms part of one of the putative operons similar to *lodAB* operon detected in the *M. mediterranea* genome analysis (Lucas-Elio et al. 2012).

Sequence comparison using the EMBOSS pairwise alignment tool (http://www.ebi.ac.uk/Tools/psa/emboss_needle/) revealed that the product of Marme_1655 shows 22.8% identity and 34.6% similarity to LodA. Although the overall similarity is not very high, it is important to point out that the product of Marme_1655 showed the conserved regions previously detected in the analysis of LodA (Lucas-Elio et al. 2006) (Fig. 4). Another relevant feature is that the product of the gene Marme_1655 shows a typical twin-arginine signal peptide (Bendtsen et al. 2005), what could be related to its detection in the supernatants of the cultures.

**Figure 5:**
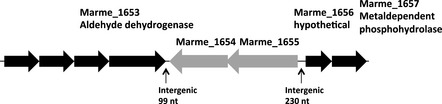
Genome region of *Marinomonas mediterranea* around gene Marme_1655.

The availability of the *M. mediterranea* genome allowed the identification of the regions surrounding Marme_1655 (Fig. 5). In the same orientation than this gene and immediately downstream is Marme_1654 which shows 26.2% identity and 43.8% similarity to LodB, a protein required for the correct expression of LodA (Gomez et al. 2010). The absence of separation between Marme_1655 and Marme_1654, and the fact that the surrounding genes are in opposite orientation indicate that they form part of the same transcriptional unit.

### Creation of a mutant with Marme_1655 deletion and complementation of the phenotype

**Figure 6:**
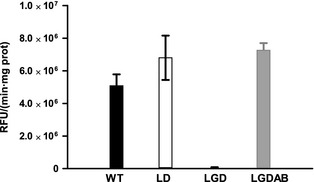
Glycine oxidase activity in supernatants of different *Marinomonas mediterranea* strains. The strains were grown for 48 h in MNGL. GOX activity is expressed as relative fluorescence units (RFU) per min and mg of protein.

The results presented in the previous section strongly support that the product of the gene Marme_1655 codes for the GO detected in the supernatants of *M. mediterranea*. To confirm this hypothesis, a mutant strain with a deletion of the genes Marme_1654 and Marme_1655, named LGD, was constructed as described in Experimental Procedures, and it is shown in Figure S1. Briefly, the protocol was based on the construction of a suicidal vector derived from pEX18Gm (Hoang et al. 1998) in which two genomic fragments located upstream and downstream of the region to be deleted were cloned. Later, the *sacB* marker in the plasmid was used to select strains that had undergone a process of double recombination. As shown in Figure S2, the method worked fine and it was possible to confirm by PCR that the operon had been deleted. This deletion determined the complete loss of GO activity (Fig. 6). Later, the plasmid pBGOXAB containing a transposon with the genes Marme_1655 and Marme_1654 plus the upstream sequence was constructed (see Experimental Procedures). This transposon was introduced into strain LGD to perform genetic complementation, generating strain LGDAB. The introduction of the two genes in the bacterial genome was confirmed by PCR (Fig. S2). It was observed that GOX activity was recovered in this strain (Fig. 6). These results clearly show that the GO detected in *M. mediterranea* is encoded by an operon that has been named *gox*, for GO activity. The two genes forming part of it have been named *goxA* (Marme_1655) and *goxB* (Marme_1654).

## Discussion

The results described in this study reveal that the Gram-negative marine bacterium *M. mediterranea* synthesizes a novel enzyme with GO activity which shows important differences with previously described GOs from the genera *Bacillus* and *Geobacillus* (Nishiya and Imanaka 1998; Martínez-Martínez et al. 2008). For instance, in terms of substrate range, the *M. mediterranea* enzyme is much more specific on glycine (Table 2). Another important difference is that *Bacillus* GO is a flavoprotein, while, on the contrary, *M. mediterranea* GoxA sequence indicates that it is an enzyme that does not use this cofactor. Based on the similarity to LodA (Gomez et al. 2010) and the sensitivity to quinoprotein inhibitors, it is proposed that it is a new example of a protein with quinone cofactor. Bioinformatic studies did not reveal any clear similarity of either LodA or GoxA to any previously described quinoprotein, supporting that they constitute a novel family of those proteins.

The *M. mediterranea* Gox is encoded by an operon containing two genes. One of them has been named *goxA* (Marme_1655) and encodes the enzyme with the oxidase activity, as revealed by SDS-PAGE and HPLC-MS/MS analysis of the peptidic fragments generated by trypsin digestion (Figs. 3 and 4). The predicted molecular mass of GoxA is 76284.80. These data suggest that in the native system, where the protein is observed as running between 130 and 170 kDa, the enzyme is probably synthesized as a dimer as it has also been proposed in the case of LodA (Lucas-Elio et al. 2005). The second gene in the operon (Marme_1654) has been named *goxB*. Based on the similarity to the *lodAB* operon, involved in the expression of the lysine-ε-oxidase of *M. mediterranea* (Gomez et al. 2010), GoxB would participate in the generation of the active form of GoxA.

Genome sequencing has revealed that different microorganisms contain genes similar to LodA (Lucas-Elio et al. 2006). Those genes are generally annotated as encoding hypothetical proteins. Genome sequencing revealed that *M. mediterranea* contains two of such homologs (Lucas-Elio et al. 2012). As already discussed, this study has shown that the product of Marme_1655 is a novel GO. One interesting issue remaining to be solved is the activity of the product encoded by the third gene (Marme_2396) as well as other similar proteins in different organisms.

On the other hand, this study has revealed in GoxA the same conserved regions (Fig. 4) that were previously detected in LodA (Lucas-Elio et al. 2006). Hopefully, structural studies will help to clarify the role of the conserved regions and residues, and the determinants of the different substrate specificity. In this regard, LodA was the first enzyme described with lysine-ε-oxidase activity (EC 1.4.3.20) (Gomez et al. 2006). This enzymatic activity was also detected in the *Pseudoalteromonas tunicata* autolytic protein AlpP (Mai-Prochnow et al. 2008). The sequence similarity between the two proteins is quite high (53.9% identity and 69.1% similarity). In addition, in *Marinomonas* sp. MWYL1, in which lysine oxidase activity has been also demonstrated (Gomez 2010), it is possible to detect a homolog (accession YP_001342557) with 72.8% identity.

Comparison between other proteins with similarity to GoxA and LodA revealed similarity values much lower, and it is not possible to make predictions about their actual enzymatic activities. For instance, two proteins from *Chromobacterium violaceum* NP_902938 and *Caulobacter crescentus* NP_419374 belonging to the same group but with lower sequence identity to LodA (29.5% and 23.1%, respectively) also play a role in biofilm development and differentiation which is mediated by hydrogen peroxide production, although their actual enzymatic activity was not described (Mai-Prochnow et al. 2008). Preliminary studies in our lab have failed to detect either GO or lysine oxidase in *C. crescentus* or *C. violaceum* (J. C. Campillo-Brocal, unpubl. results). One possibility is that those genes were not expressed under the growth conditions used. Another possibility is that the products of those genes encode different enzymatic activities that remain to be determined. This should also be the case of the product of the gene Marme_2396 assuming that it is expressed in *M. mediterranea* LGD, with deletion of *lod* and *gox* operons, and that lacks both lysine and GO activities.

The results presented in this study strongly suggest that the group of proteins with different degrees of similarity to LodA and GoxA detected in bacterial genomes constitute a reservoir of different enzymatic activities to be explored and characterized. As the two members characterized up to now, show amino acid oxidase activity, a possibility could be that the others could also oxidize different amino acids. However, it is important to take into consideration that the amino group oxidized in lysine is in epsilon position, and although glycine is oxidized in alpha position, this is the smallest aminoacid. Accordingly, it cannot be ruled out that other kinds of substrates could be oxidized by some of the other enzymes.

## Supporting Information

Figure S1 Construction of mutant strain with a deletion of the *gox* operon. (A) Scheme showing the plasmid constructed and the genomic region around the *gox* operon. The two fragments flanking the operon cloned in the plasmid are colored in red and blue, respectively. (B). Representation of the genomic region after plasmid insertion by homologous recombination. The integration could take place in any of the two regions. (C) Possible outcomes after a second recombination event with loss of the integrated plasmid.

Figure S2 Determination by PCR of the presence or absence of the *gox* operon. (A) Scheme of the genome region around Marme_1655 in the wild-type strain (1) and in the deletion strain (2). The regions upstream and downstream used to generate the deletion, as well as the position of the primers are marked. (B) Confirmation by PCR of the deletion of *goxAB*. 1: PCR with internal primers (GOSEC1-GOREVSEC2); 2: PCR with external primers (GOXDIRSAC-GOXREVPST; in the PCR conditions it gives no product in the wild type. (C) Confirmation by PCR with internal primers of the reintroduction of operon *goxAB* in strain LGDA.
