# Supplementary material for: Identification in Marinomonas mediterranea of a novel quinoprotein with glycine oxidase activity
Source: Microbiologyopen. 2013 Jul 22;2(4):684–94. doi: 10.1002/mbo3.107 (PMC3948610; doi:10.1002/mbo3.107)
Supplement: Figure S1 — Construction of mutant strain with a deletion of the gox operon. (A) Scheme showing the plasmid constructed and the genomic region around the gox operon. The two fragments flanking the operon cloned in the plasmid are colored in red and blue, respectively. (B). Representation of the genomic region after plasmid insertion by homologous recombination. The integration could take place in any of the two regions. (C) Possible outcomes after a second recombination event with loss of the integrated plasmid. [file mbo30002-0684-sd1.pdf]

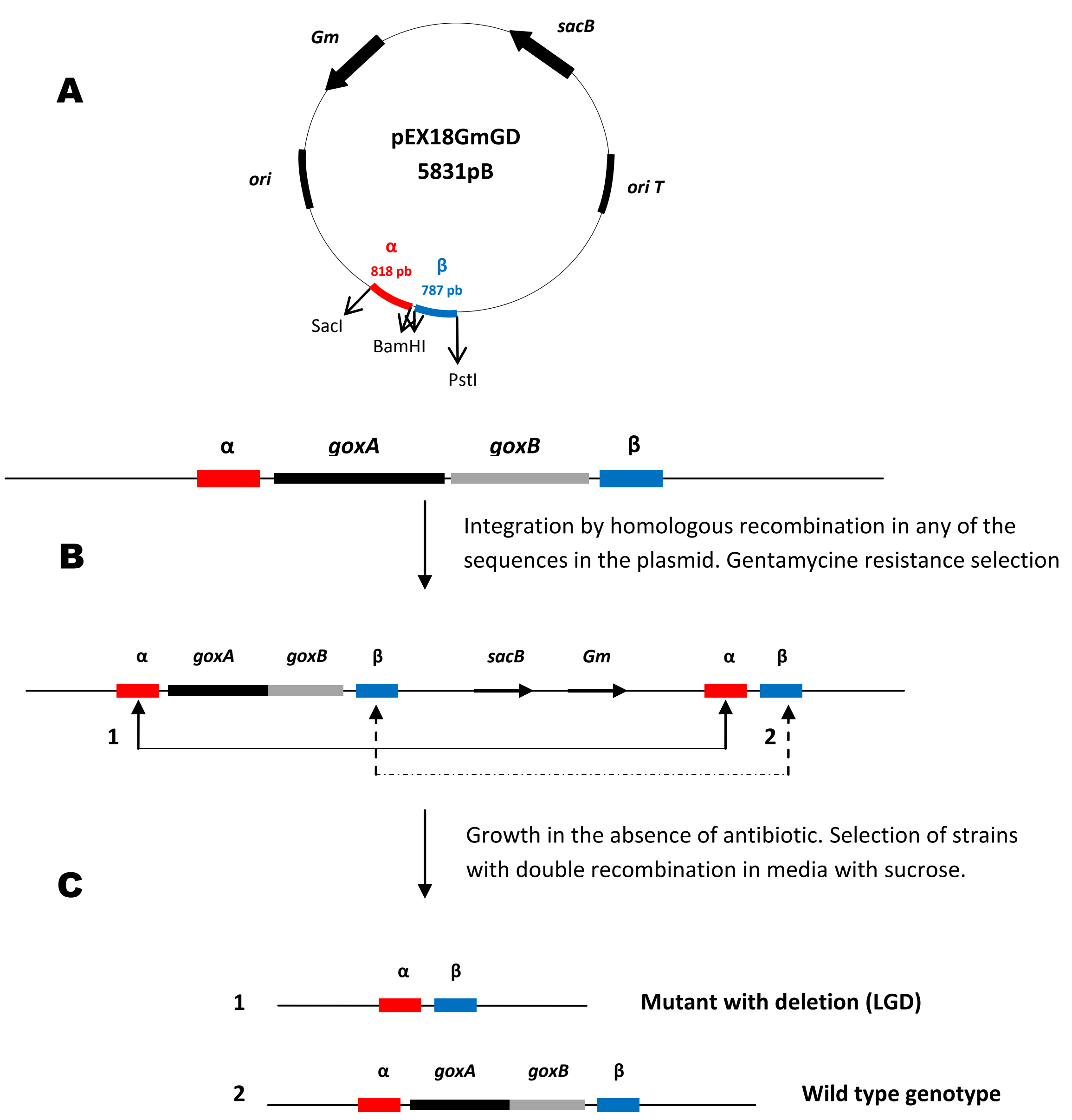

Fig. S1. Construction of mutant strain with a deletion of the *gox* operon. (A) Scheme showing the plasmid constructed and the genomic region around the gox operon. The two fragments flanking the operon cloned in the plasmid are coloured in red and blue respectively. (B). Representation of the genomic region after plasmid insertion by homologous recombination. The integration could take place in any of the two regions. (C) Possible outcomes after a second recombination event with loss of the integrated plasmid.

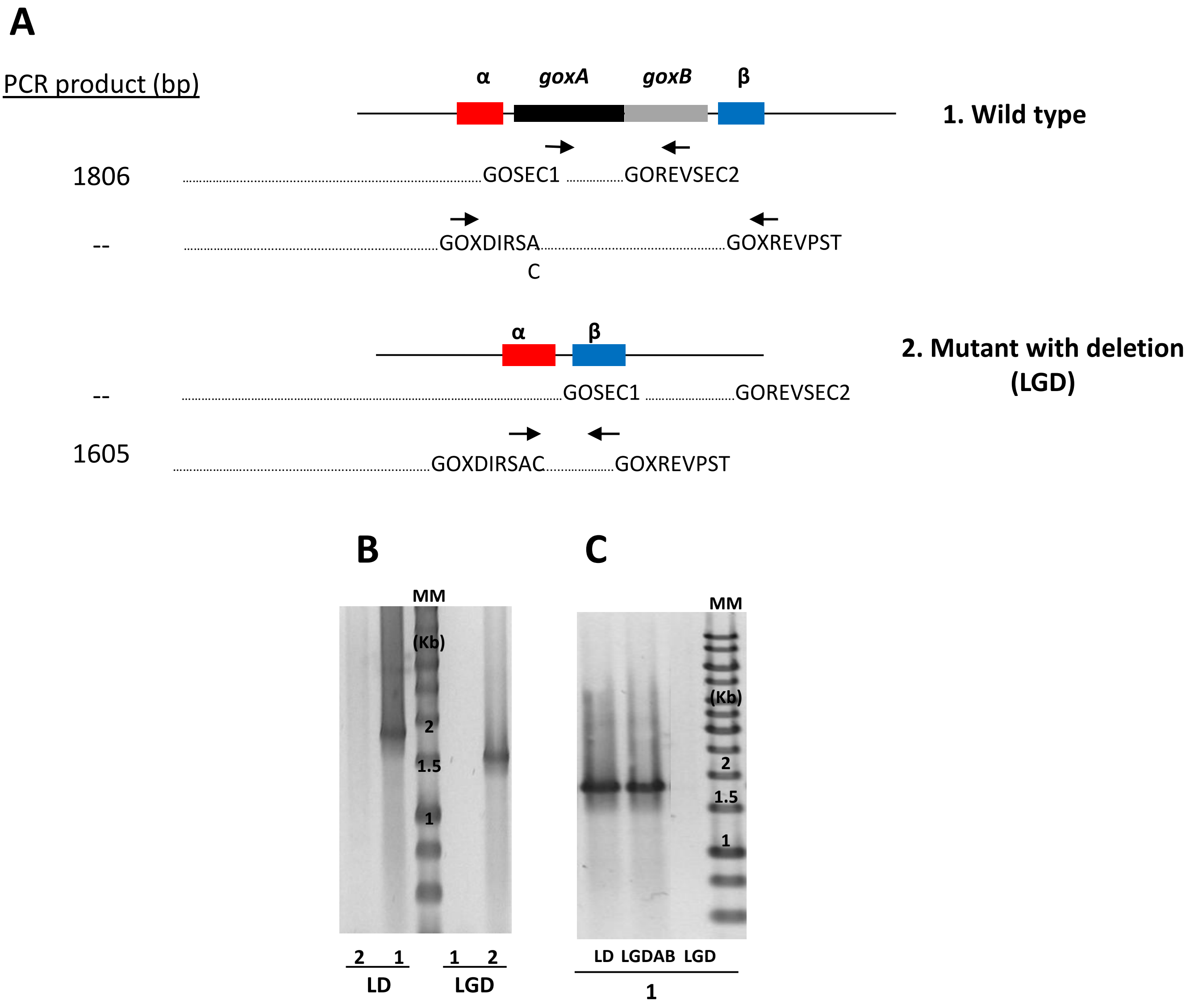

Fig. S2. Determination by PCR of the presence or absence of the *gox* operon . **(**A) Scheme of the genome region around Marme_1655 in the wild type strain (1) and in the deletion strain (2). The regions upstream and downstream used to generate the deletion, as well as the position of the primers are marked. (B) Confirmation by PCR of the deletion of *goxAB*. 1: PCR with internal primers (GOSEC1-GOREVSEC2); 2: PCR with external primers (GOXDIRSAC-GOXREVPST; in the PCR conditions it gives no product in the wild type (C) Confirmation by PCR with internal primers of the re-introduction of operon *goxAB* in strain LGDA.
